# Increased signals from short-wavelength-excited fluorescent molecules using sub-Ti:Sapphire wavelengths

**DOI:** 10.1111/j.1365-2818.2012.03663.x

**Published:** 2012-11

**Authors:** G NORRIS, R AMOR, J DEMPSTER, W B AMOS, G MCCONNELL

**Affiliations:** * Centre for Biophotonics, SIPBS, University of StrathclydeGlasgow, U.K.; † MRC Laboratory of Molecular BiologyCambridge, U.K.

**Keywords:** Autofluorescence, live cell, multiphoton, short-wavelength

## Abstract

We report the use of an all-solid-state ultrashort pulsed source specifically for two-photon microscopy at wavelengths shorter than those of the conventional Ti:Sapphire laser. Our approach involves sum–frequency mixing of the output from an optical parametric oscillator (λ= 1400–1640 nm) synchronously pumped by a Yb-doped fibre laser (λ= 1064 nm), with the residual pump radiation. This generated an fs-pulsed output tunable in the red spectral region (λ= 620–636 nm, ∼150 mW, 405 fs, 80 MHz, *M*^2^∼ 1.3). We demonstrate the performance of our ultrashort pulsed system using fluorescently labelled and autofluorescent tissue, and compare with conventional Ti:Sapphire excitation. We observe a more than 3-fold increase in fluorescence signal intensity using our visible laser source in comparison with the Ti:Sapphire laser for two-photon excitation at equal illumination peak powers of 1.16 kW or less.

## Introduction

The ultrashort pulsed Ti:Sapphire laser has proven highly successful in producing multiphoton excitation of fluorochromes and autofluorescent materials (Curley *et al.*, 1992; Piston *et al.*, 1995; Wu & Qu, 2005; Uchugonova & Koenig, 2008). This laser can deliver high average powers with pulse durations routinely shorter than 300 fs and wavelength tuning over λ= 680–1080 nm. The wide tuning range allows the user to match the wavelength of the laser to the two-photon absorption peak of the molecule of interest, either empirically or by consideration of the two-photon excitation spectrum, if this is available (Lacowicz, 1999). However, although the wavelength tuning range of the Ti:Sapphire laser is broad, it is too weak for effective excitation at short wavelengths which are of potential value.

There exist many synthetic and naturally occurring fluorescent molecules with sub-λ= 360 nm single-photon excitation peaks, including Indo-1, 4’,6-diamidino-2-phenylindole (DAPI) Hoechst Blue, NADH, tryptophan and phenylalanine (Sako *et al.*, 1997; Peng *et al.*, 2004; Tsien *et al.*, 2006). Single-photon excitation could be applied for these molecules, with current diode technology allowing this to be done at low cost (McGuinness *et al*., 2004). However, intrinsic issues with working in the ultraviolet (UV) range, that is, sample damage and the requirement for specialized UV optics, make this approach unattractive. Two-photon excitation offers a route to by-pass this harmful spectral range and can be applied to many commercial microscopes. However, due to the lack of inexpensive ultrashort pulsed laser sources at wavelengths shorter than those of the Ti:Sapphire, little is known of the two-photon excitation dynamics at wavelengths between λ= 600 and 650 nm. Such two-photon Ti–sapphire excitation spectra that are available show the two-photon excitation cross-section increasing towards shorter excitation wavelengths (Xu *et al.*, 1996; Xu & Webb, 1996; Albota *et al.*, 1998; Huang *et al.*, 2002).

Efforts have been made to provide ultrashort pulsed output in this spectral region. Dye lasers can be made to work, as was demonstrated by Denk *et al.* in their seminal paper on two-photon microscopy (Denk *et al*., 1990). However, problems with these lasers include instability, while the often toxic gain material requires careful handling upon replacement, as it deteriorates in time (Masters & So 2004). One group has demonstrated a photonic crystal fibre-based light source for two-photon excitation of tryptophan powder at wavelengths between λ= 500 nm and λ= 600 nm (Palero *et al.*, 2005). However, the system seems never to have been applied to live biological specimens. This is perhaps because of the long image acquisition times required (>2 min per image) or the low fluorescence signal that can be obtained with such a source at normal biological concentrations of tryptophan. The frequency-doubled Cr:Forsterite laser is another potential candidate for ultrashort pulsed radiation in the red spectral range, but the present cost of this system means that it is beyond the budget of most. The same applies to an optical parametric oscillator system pumped by a frequency-doubled Ti:Sapphire laser. This latter approach is attractive, insofar as many research laboratories engaging in two-photon microscopy already have access to such a laser, but the additional cost of a commercial frequency doubler and a commercial optical parametric oscillator is high. It is also noted that three-photon excitation through the use of a Ti:Sapphire laser is possible for imaging of UV absorbing fluorophores (Gryczynski *et al*., 1995). However, the three-photon cross-section of many dyes are very low and require very high levels of peak power, which can be detrimental to the health of the sample.

We report here a two-step nonlinear optical frequency conversion system as the basis of an all-solid-state ultrashort pulsed source specifically for two-photon microscopy at wavelengths shorter than the Ti:Sapphire. Our approach involves a single pump laser split into two beams, the first used to synchronously pump an optical parametric oscillator, the output of which is sum–frequency mixed with the remaining pump beam. This novel laser system has a brightly visible red emission, which tends to make the beam safer to use than the deceptively dull red of even the most powerful Ti:Sapphire beam. We demonstrate the performance of our ultrashort pulsed system using fluorescently labelled tissue and naturally occurring fluorophores, and perform a comparison with conventional Ti:Sapphire excitation. In the first specimen, namely fixed mouse kidney labelled with the DNA stain DAPI, the same structures (cell nuclei) were visible using both lasers, but more than a 3-fold increase in fluorescence signal was obtained using the visible-light laser source. We also imaged a live sample of a leaf of a green plant (*Hosta* sp), with the aim of visualising the chlorophyll-rich guard cells. Using the Ti:Sapphire laser the guard cells yielded low fluorescence, but the ultrashort pulsed red laser provided clear images, without inducing damage to the specimen. We note that the total cost of both laser platform and microscope is less than that of a single ultrashort pulsed Ti:Sapphire laser.

## Experimental set-up

**Fig. 1:**
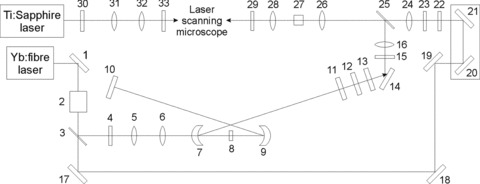
A schematic diagram of the optical parametric oscillator (OPO) system and Ti:Sapphire beam paths used to generate ultrashort pulsed red light via sum–frequency mixing (SFM). Elements 1 and 17–21 are highly reflecting plane mirrors at the pump wavelength of λ= 1064 nm. Element 2 is a Faraday isolator. Element 3 is a 50/50 thin-plate beamsplitter at λ= 1064 nm. Element 4 is a half-wave plate at λ= 1064 nm. Elements 5 & 6 are mode-matching lenses. Elements 7 & 9 are zero lens ROC = 100 mm mirrors (zero lens mirrors have the same radius of curvature on both surfaces, so a beam propagating through the element is not focused or defocused, making it easier to design the optical system and control the beam), highly transmitting at λ= 1064 nm and highly reflecting at λ= 1500 nm. Element 8 is a 3-mm-long PPLN crystal; 11 is a 50% reflectivity mirror at λ= 1500 nm, which serves as the OPO output coupler. Element 12 is a half-wave plate at λ= 1500 nm and 13 is a variable neutral density filter. Element 14 is a high reflectivity plane mirror at λ= 1500 nm, and 15 is a long-wave pass filter. Element 22 is a variable neutral density filter for use at λ= 1064 nm; 23 is a half-wave plate at λ= 1064 nm and 24 is a lens. Element 25 is a high-reflectivity plane mirror at λ= 1500 nm that is highly transmitting at λ= 1064 nm; 26 is a lens; 27 is the PPLN crystal for SFM of λ= 1064 nm and the OPO. Element 28 is a collimating lens and 29 is a half-wave plate for use with visible wavelengths. Element 30 is a half-wave plate for use with the Ti:Sapphire wavelengths. Elements 31 and 32 are lenses to shape the beam and 33 is a neutral density filter for use with the Ti:Sapphire laser.

The schematic diagram of the nonlinear optical system is shown in Figure 1.

A continuous wave mode-locked Yb-doped fibre laser served as the pump source, with an average power of 2 W and repetition rate of 80 MHz, pulses of 260 fs duration at a wavelength of λ= 1064 nm and with a spectral width of 12 nm full-width at half maximum (FWHM) (Fianium, Femtopower 1060–2-s). This laser was split into two components by a 50/50 beamsplitter (element 3). The first beam was used to synchronously pump an optical parametric oscillator (OPO), the output of which was recombined spatially and temporally with the second pump beam in a second nonlinear optical crystal to perform sum–frequency mixing (SFM).

We first consider the OPO. The basic OPO has already been described (Norris & McConnell, 2010) and for ease, a single pump pass, singly resonant OPO was chosen, with the signal wavelength resonant. This source provided a 260 fs pulsed pump source which was continuously tunable between 1400 and 1650 nm with an output power of ∼200 mW.

The output from the OPO was then propagated through a variable neutral density filter (element 12) and a long-pass filter [Thorlabs (Ely, UK) FB1400–12 (element 13)] to ensure that residual pump wavelength radiation at λ= 1064 nm was blocked and only the signal wavelength output at λ > 1400 nm was available for the SFM process. Element 14 was a highly reflecting mirror at the signal wavelength and elements 15 and 16 were a half-wave plate to control the polarization of the OPO and an *f*=+80 mm convex lens to facilitate beam-shaping of the signal wavelength output prior to the SFM process.

The benefit of using an OPO as one of the pump sources for SFM was the ability to vary the wavelength and to optimize the phase-matching conditions. In conjunction with the control of the temperature of the SFM periodically poled lithium niobate (PPLN) crystal, the wavelength of the output could be altered while maintaining a zero phase mismatch.

We now consider the second pump beam used for the SFM process, Elements 17, 18 and 19 were used to steer the beam towards a trombone arrangement. This comprised two highly reflective dielectric mirrors at the pump wavelength, which were mounted on a single linear translation stage (elements 20 and 21). This created a time delay, permitting temporal displacement of the pump pulse, which was used to synchronize the pump pulse and signal wavelength pulse from the OPO within the nonlinear material for SFM. Elements 21 and 22 were variable neutral density filter to control the intensity of the radiation reaching the SFM arrangement and an *f*=+100 mm lens to shape the signal wavelength output prior to the SFM process.

The pump and signal wavelength beams were combined at element 23, which was a plane mirror highly reflecting at the signal wavelength and highly transmitting at the pump wavelength, similar to element 10, used as a cavity end mirror in the OPO. The two beams were then spatially and temporally overlapped and focused using a single *f*=+25 mm spherical lens, antireflection coated for at λ= 1000–1600 nm for minimal power loss (element 24). This focused both beams into a 1-mm-long PPLN crystal, this time with a domain period length Λ= 11.12–11.22 μm, designed for SFM of the pump and signal wavelengths. The sum–frequency mixed output was then collimated using an *f*=+25 mm spherical lens, this time antireflection coated at λ= 400–800 nm (element 26). Element 27 was a λ= 700 nm short-wave pass filter (ET700sp-2p8, Chroma Technology, Bellows Falls, VT, U.S.A.) to block the pump and signal wavelength outputs, and transmit the sum–frequency mixed wavelength only. This was then directed towards a home-built laser scanning microscope.

For comparison with existing technology, the output from a commercial ultrashort pulsed Ti:Sapphire laser (Mira 900-F, Coherent, Santa Clara, CA, U.S.A.) was also coupled into the laser scanning microscope. The polarization of the Ti:Sapphire laser was fine-tuned using a half-wave plate designed for operation at wavelengths λ= 700–1050 nm (element 30), and the beam shaped using spherical lenses of *f*=+100 mm and *f*=+150 mm (elements 31 and 32) to match the beam profile of the sum–frequency mixed source. The average power of the Ti:Sapphire laser exceeded 200 mW across the tuning range, and so a variable neutral density filter (element 33) was used to ensure the delivery of the same peak intensity to the specimen as was provided by the sum–frequency mixed source. All optical properties of the sum–frequency mixed and Ti:Sapphire lasers were identical where possible, including, importantly the peak power at the specimen plane. The Ti:Sapphire was operated at a wavelength of λ= 720 nm and gave a pulse duration of 250 fs at a repetition rate of 76 MHz. The Ti:Sapphire beam was expanded to provide a similar beam diameter to the TEM00 sum–frequency mixed output to fill the back aperture of the objective lens.

We have assumed that the higher-order dispersion effects in the microscope system were negligible and equivalent for the two wavelengths used, on the grounds that the >200 fs pulse duration would virtually eliminate such effects.

Two mirrors and a periscope system were used to steer the sum–frequency mixed or Ti:Sapphire radiation towards a home-built laser scanning microscope. This is described elsewhere in detail (Norris *et al.*, 2012). A 20×/0.75 N.A. dry objective lens (Nikon Plan Fluor, Nikon, UK) was used and the percentage transmission for the Ti:Sapphire and sum–frequency mixed sources were equivalent to within 5% across the available wavelength ranges. A condenser lens of N.A. = 0.9 was used in the transmission path to collect the fluorescence or autofluorescence signals. In these experiments we used a single λ= 700 nm short-pass filter (et700sp-2p8, Chroma, Bellows Falls, VT, USA) and a λ= 550 ± 40 nm bandpass filter (NT65–704, Edmund Optics Ltd., York, U.K.) in the transmission path to block the excitation wavelength and any harmonic generation, such that only fluorescence and autofluorescence from the specimens was transmitted. A photomultiplier tube (RFI-QL-30F, Thorn EMI, London, U.K.) was employed to collect the nondescanned fluorescence signal. To control the scanning galvo mirrors and for image capture, we used the freely available MPScope software (Nguyen *et al.*, 2006). For all experiments, the image size was 512 × 512 pixels taken at 2.18 frames per second, with a pixel dwell time of 1.4 μs.

## Results

**Fig. 2:**
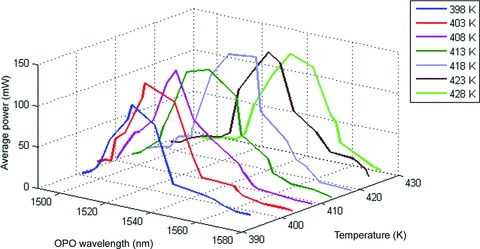
Average powers over the tuning range of the sum–frequency mixed wavelengths with respect to the sum–frequency mixing (SFM) PPLN crystal temperature (K) and the optical parametric oscillator (OPO) signal wavelength.

Our apparatus gave a sum–frequency mixed wavelength tunable from λ= 620–636 nm. Figure 2 shows the average output power corresponding to input OPO wavelength while varying the temperature of the SFM PPLN crystal.

The maximum average output power from the SFM process was 150 mW at a wavelength of λ= 630 nm with a beam quality of *M*^2^∼ 1.3. This condition corresponded to mixing of the λ= 1064 nm output from the pump laser with λ= 1538 nm radiation from the OPO, and an SFM PPLN crystal temperature of 418 K with a period length of λ= 11.7 μm. Tuning of the OPO signal wavelength was achieved by a combination of temperature tuning and OPO PPLN period length selection. Signal wavelengths below λ= 1500 nm and above λ= 1570 nm generated negligible powers. This was also the case for SFM PPLN crystal temperatures below 398 K and above 428 K.

**Fig. 3:**
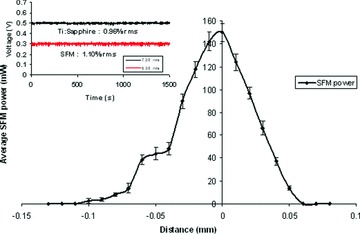
Average power at a sum-frequency mixed wavelength of λ= 630 nm (1064 nm + 1550 nm) as a function of temporal overlap of the two input wavelengths within the SFM PPLN crystal. Inset: Comparison of the stability of the sum–frequency mixing (SFM) source, measured at the peak output power, and the commercial Ti:Sapphire laser.

By controlling the length of the delay line (relative positioning of elements 20 and 21 to elements 19 and 22 in Fig. 1), at a fixed wavelength of λ= 630 nm the FWHM range of overlap was found to span over approximately 60 μm. This is shown in Figure 3, along with inset stability measurements for both the SFM source and the Ti:Sapphire laser. The stability of the SFM source (1.1% rms) compares favourably with the commercial source (0.96% rms). Over the detuning range, pulse durations were measured, using an autocorrelator (APE, Pulsecheck, Fremont, CA, U.S.A.), to be 405 ± 15 fs.

**Fig. 4:**
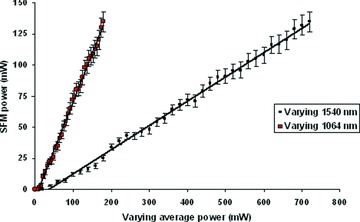
Sum–frequency mixed average power as a function of λ= 1064 nm pump power, with optical parametric oscillator (OPO) power at λ= 1550 nm fixed at 200 mW and sum-frequency mixed average power as a function of λ= 1550 nm OPO power, with the pump power at λ= 1064 nm fixed at 750 mW. Each point averaged over an *n*= 10.

By the use of variable neutral density filters to control independently the applied power from the pump and OPO to the SFM PPLN crystal, it was possible to determine the dependence of the sum–frequency mixed average power upon each source. This is shown in Figure 4, where either the pump or OPO beam was held at fixed average power and the other was increased incrementally. In both cases, a linear dependence was observed, as was expected for the nonlinear SFM process.

To test our system, we imaged both fixed and live biological specimens. The fixed specimen was a thin section (16 μm) of mouse kidney labelled with DAPI (Invitrogen). DAPI was used because it is a common and well-characterized two-photon fluorochrome for the Ti–sapphire laser, and one in which the characteristic nuclear localization gives reassurance that it is the same dye that is excited by both lasers when comparative measurements are made. The second specimen was a live preparation of *Hosta* sp leaf lower epidermis, with a thickness of less than 100 μm. The plant leaf was chosen to test whether the short wavelength of the new laser would show a characteristic fluorescence pattern in spite of the complexity of the living material. We chose this specimen to test whether short-wavelength two-photon excitation has any useful specificity, in view of the possibility that tyrosine and tryptophan fluorescence might make almost all proteins fluoresce at these novel wavelengths, as happens with single-photon short-wavelength UV excitation. We note that both of the samples used in our analysis were relatively thin, if thicker tissues were to be imaged, it would be advisable to couple our system with a method of predispersion compensation to compensate for any pulse broadening incurred within a sample, such as that provided by current commercial systems.

**Fig. 5:**
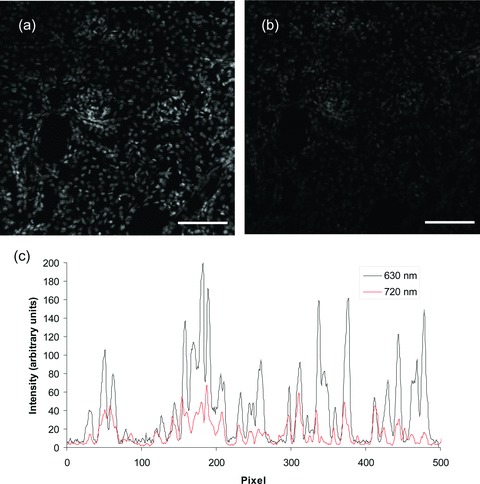
(a) Fluorescence image of cell nuclei labelled with 4’,6-diamidino-2-phenylindole (DAPI) in a fixed section of mouse kidney using the sum–frequency mixed λ= 630 nm source for two-photon excitation. (b) Fluorescence image the mouse kidney section using a Ti:Sapphire laser source at a wavelength of λ= 720 nm source for two-photon excitation. (c) A typical example of an intensity line scan using ImageJ to analyse the fluorescence signal intensity from comparable regions. In excess of a 3-fold increase in intensity is consistently observed using the red light at λ= 630 nm in comparison with the λ= 720 nm Ti:Sapphire laser for two-photon excitation. The scale bars correspond to 50 μm.

Figure 5 shows two-photon excitation images of the mouse kidney specimen using (a) the sum–frequency mixed source at λ= 630 nm and (b) the Ti:Sapphire laser at λ= 720 nm. The peak power at the specimen plane used to acquire both images (a) and (b) was 1.16 kW. Line plots of the fluorescence intensity greyscale values (8 bits) were measured for each image (*n*= 10 images) using both the 630 nm source and 720 nm Ti:Sapphire laser for excitation of the fluorophore. A comparison of the fluorescence signal intensity is shown in Figure 5(c). We consistently observe more than a 3-fold increase in detected fluorescence signal using the sum–frequency mixed source in comparison with the longer wavelength Ti:Sapphire laser when using the same peak power of illumination from both sources. As a measure of significance for this result, a *t*-test was performed and a *p*-value was determined. As this *p*-value of 4.9 × 10^−6^ falls well below the accepted indicator of significance (0.05), we conclude that this is a true assessment.

**Fig. 6:**
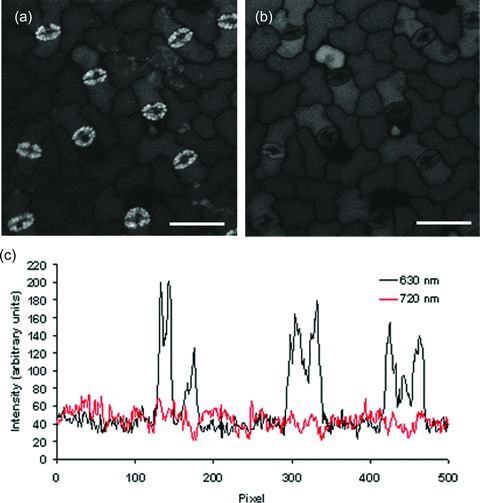
(a) Fluorescence image of live *Hosta* sp leaf epidermis using the sum–frequency mixed λ= 630 nm source for two-photon excitation. (b) Fluorescence image of the same region of live *Hosta* sp using a Ti:Sapphire laser source at a wavelength of λ= 720 nm source for two-photon excitation. In (a) the stomata are clearly visible because of guard cell autofluorescence, whereas in (b) there is higher background but less specificity. (c) A typical example of an intensity line scan passing through three guard cells was extracted using ImageJ. It was found that regions containing guard cells in *n*= 10 images, more than a 3-fold increase in fluorescence signal intensity was observed when using the red light at λ= 630 nm in comparison with the λ= 720 nm Ti:Sapphire laser for two-photon excitation. Scale bars correspond to 50 μm.

Figure 6 shows two-photon excitation images of the *Hosta* sp leaf epidermis showing autofluorescence. Figure 6(a) was captured using the sum–frequency mixed source at λ= 630 nm and Figure 6(b) was obtained using the Ti:Sapphire laser source at λ= 720 nm. The peak power used to acquire the images was 0.95 kW, with the same peak power provided at the specimen plane from the two sources. Using ImageJ to analyse the fluorescence signal intensity from comparable line scans containing the guard cells in *n*= 10 images, we observed more than a 3-fold increase in fluorescence signal intensity using the red light at λ= 630 nm in comparison with the λ= 720 nm Ti:Sapphire laser for two-photon excitation. With the Ti:Sapphire laser source, there was a slightly higher level of background autofluorescence but the stomata are not clearly visible. However, the shorter-wavelength sum–frequency mixed source gave a much improved signal to background ratio for studying the stomata present within the specimen. The autofluorescence seems likely to be due to chlorophyll. In view of the finding that chlorophylls emit in the blue after two-photon excitation at λ= 650–680 nm (Leupold *et al.*, 2002) it would be of interest to study the fluorescence of chlorophyll at the shorter wavelengths supplied by our laser.

## Conclusion

We conclude from our study that SFM, which can provide wavelengths shorter than those of the Ti:Sapphire laser source, can increase the fluorescence signal intensity from biological specimens at similar illumination powers. Our approach involves SFM of the output from a synchronously pumped OPO with residual pump radiation to generate fs-pulsed output in the red spectral region. We demonstrated the performance of our ultrashort pulsed system using fluorescently labelled tissue and naturally occurring fluorochromes, and, in a comparison with a conventional Ti:Sapphire laser source, we observed more than a 3-fold increase fluorescence signal intensity using our visible laser source in both the DAPI-labelled mouse kidney and the *Hosta* sp leaf epidermis. This alternative to the Ti:Sapphire laser for short-wavelength excitation offers the possibility of reducing the power at the specimen, which may lead to an important reduction in photo-damage of biological tissue

Our approach to two-photon imaging also has great potential as a replacement for other UV techniques. This includes UV fluorescence lifetime imaging (Li *et al*., 2004; Schneckenburger *et al*., 2004) which may increase the viability of the specimen. Also, monitoring of enzyme activity based upon fluorescence anisotropy measurements via spectrally and polarization-resolved imaging, as presented by Bigelow *et al.* (2004) may benefit. Here, two-photon-excited anisotropy would provide the added advantage of an enhanced dynamic range, yielding additional information from the images recorded. Both of these proposed applications highlight the widespread impact which is possible from our excitation source.

Finally, we note that imaging based upon two-colour two-photon excitation within a specimen (equivalent to SFM) is possible and has been previously demonstrated (Quentmeier *et al*. 2009). However, due to reduced control of the SFM process and dispersion-related issues when using two beams of different wavelengths within a sample, imaging becomes challenging. Also, one of the chosen wavelengths for this process is required to be short (400 nm in the case of Quentmeier), which is a harmful spectral range for biological samples to be exposed to, hence negating one of the fundamental benefits of two-photon excitation. Therefore we see SFM prior to the sample as a much more straightforward and beneficial imaging technique.
